# Mesenchymal Stem Cell Secretome: Potential Applications in Human Infertility Caused by Hormonal Imbalance, External Damage, or Immune Factors

**DOI:** 10.3390/biomedicines13030586

**Published:** 2025-02-27

**Authors:** Katerina Kavaldzhieva, Nikola Mladenov, Maya Markova, Kalina Belemezova

**Affiliations:** Department of Biology, Medical Faculty, Medical University of Sofia, 1431 Sofia, Bulgaria; kkavaljieva@medfac.mu-sofia.bg (K.K.); n.mladenov@medfac.mu-sofia.bg (N.M.); m.markova@medfac.mu-sofia.bg (M.M.)

**Keywords:** mesenchymal stem cells, secretome, exosomes, infertility, tissue repair and regeneration, immunomodulatory functions, anti-apoptotic activity, paracrine signaling

## Abstract

Mesenchymal stem cells (MSCs) are a source of a wide range of soluble factors, including different proteins, growth factors, cytokines, chemokines, and DNA and RNA molecules, in addition to numerous secondary metabolites and byproducts of their metabolism. MSC secretome can be formally divided into secretory and vesicular parts, both of which are very important for intercellular communication and are involved in processes such as angiogenesis, proliferation, and immunomodulation. Exosomes are thought to have the same content and function as the MSCs from which they are derived, but they also have a number of advantages over stem cells, including low immunogenicity, unaltered functional activity during freezing and thawing, and a lack of tumor formation. In addition, MSC pre-treatment with various inflammatory factors or hypoxia can alter their secretomes so that it can be modified into a more effective treatment. Paracrine factors secreted by MSCs improve the survival of other cell populations by several mechanisms, including immunomodulatory (mostly anti-inflammatory) activity and anti-apoptotic activity partly based on Hsp27 upregulation. Reproductive medicine is one of the fields in which this cell-free approach has been extensively researched. This review presents the possible applications and challenges of using MSC secretome in the treatment of infertility. MSCs and their secretions have been shown to have beneficial effects in various models of female and male infertility resulting from toxic damage, endocrine disorders, trauma, infectious agents, and autoimmune origin.

## 1. Introduction

Since the beginning of the twenty-first century, the field of stem cell research has expanded incredibly quickly, and numerous novel findings have emerged that highlight the most fascinating aspects of biological and medicinal knowledge. Mesenchymal stem cells (MSCs) have attracted great interest among scientists in recent years for several reasons. They have the potential for self-renewal and can be obtained from a variety of sources, such as bone marrow, adipose tissue, umbilical cord tissue, skin, muscle, and dental pulp. They also possess in vitro differentiation capacity and can transdifferentiate into various cell types, including neural cells, insulin-producing β cells, and hepatocytes. The ethical issues regarding the isolation and use of MSCs (discussed below) are far easier to address than those associated with other treatment approaches, which further encourages scientists to seek appropriate ways to use these cells to treat various diseases. However, recent studies have increasingly demonstrated that the most promising therapeutic potential of MSCs is hidden in the factors and vesicles they secrete, known as secretome.

## 2. Stem Cells

Stem cells are clonogenic cells possessing two remarkable properties that allow them to maintain tissue homeostasis: the ability to differentiate into different cell types [1] and the ability to undergo multiple cycles of cell division while maintaining their undifferentiated state (self-renewal) [2]. Stem cell renewal can be symmetrical (generating two stem cells) or asymmetrical, which is one of the most fundamental properties of adult stem cells, guaranteeing that one daughter cell becomes committed to differentiation, while the other daughter cell retains its stem cell identity [3]. The ability of a stem cell to differentiate into different lineages is known as potency [4]. It dictates what is probably the most significant characteristic of stem cells: their capacity to produce mature, functional cell types in tissues and organs that are essential to the organism’s survival. According to this potential, stem cells are divided into totipotent, pluripotent, multipotent, oligopotent, and unipotent. Totipotency is defined as the ability of a cell to differentiate into both embryonic and extraembryonic tissues. Totipotent stem cells are produced immediately after the fusion of the egg and sperm and, in mammals, are generated within the first few cell divisions after fertilization. Cells are pluripotent if they have the ability to differentiate into all three primary germ layers (endoderm, mesoderm, and ectoderm) but cannot contribute to extraembryonic tissues. Pluripotent stem cells are embryonic stem cells (ESCs) and embryonic germ cells (EGCs) isolated respectively from the inner cell mass of the blastocyst and the primordial germ cells of an early embryo. Multipotent stem cells can differentiate into a limited number of cells originating from a single germ layer. These cells are usually located in protected tissue spaces called niches and are in the G_0_ phase of the cell cycle [5]. As they are frequently required to replace distinct types of differentiated cells that deteriorate over time, multipotent cells are common in adult tissues. Hematopoietic stem cells (HSCs), which develop into diverse kinds of adult blood cells, and mesenchymal stem cells, which produce differentiated chondrocytes, osteocytes, and adipocytes, are two examples of multipotent stem cells. According to the stage of development of the organism from which they originate, stem cells are divided into embryonic stem cells, embryonic germ cells, fetal stem cells, and somatic stem cells.

## 3. Mesenchymal Stem Cells

Mesenchymal stem cells are a type of postembryonic stem cells that can be isolated from various tissues and organs of an adult individual. Such cells have been isolated and characterized from bone marrow [6], umbilical cord blood, the central nervous system [7], skin [8], muscle [9], placenta [10], hair follicles [11], etc. It is important to note that MSCs isolated from different sources are not the same. Factors such as the source and isolation methodology can affect the morphology and function of the isolated cells. The International Society for Cellular Therapy (ISCT) published minimal criteria for defining human mesenchymal stem cells, which included adherence to plastic, specific expression of surface antigens (CD73, CD90, and CD105), lack of expression of CD45, CD11b, CD14, CD19, CD34, CD79a, and HLA-DR, and the potential for multipotent differentiation [12]. However, there is still no set of straightforward, widely agreed standards that define MSCs. It is important to mention that in 2016, ISCT issued a statement emphasizing that culture conditions can affect MSC functions and, consequently, their clinical application should be closely studied [13].

As MSCs are found in the perivascular spaces, they can reach almost every tissue, which enables them to interact with a variety of cell types and take part in tissue regeneration processes. The immunomodulatory properties of MSCs are arguably the most fascinating yet poorly understood feature of their nature. NK cells, dendritic cells, T and B cells, and macrophages are among the immune cells whose functions are modulated by MSCs. MSCs adopt an immunosuppressive phenotype when they are activated in an inflammatory environment with elevated amounts of proinflammatory cytokines, including TNFα, IL-1β, and IFNγ [14].

MSCs are commonly administered to treat diseases by systemic infusion. Although this method results in increased efficacy of treatment, there is little direct evidence that MSCs differentiate into damaged tissue cells. Horwitz and Dominici summarized the role of cytokine secretion by MSCs in tissue regeneration [15], and subsequent studies have increasingly focused on the secretome of MSCs because of its functions in immunomodulation, tissue healing, and anti-inflammatory responses [16].

## 4. Molecular Mechanisms Behind the Effect of MSC-Derived Secretome

The term “stem cell secretome” refers to all of the soluble factors that stem cells produce and use for signaling between cells. The secretome consists of a wide range of serum proteins, growth factors, angiogenic factors, hormones, cytokines, chemokines, extracellular matrix proteins, and even, in small amounts, genetic material and lipid mediators [17]. These secreted molecules are released from stem cells by classical and nonclassical mechanisms of secretion, including protein translocation, exocytosis, and encapsulation of vesicles or exosomes. Soluble factors and vesicles secreted by stem cells can act directly by mediating intracellular pathways in damaged cells or indirectly by inducing the secretion of functionally active products from neighboring tissues [18].

**Figure 1:**
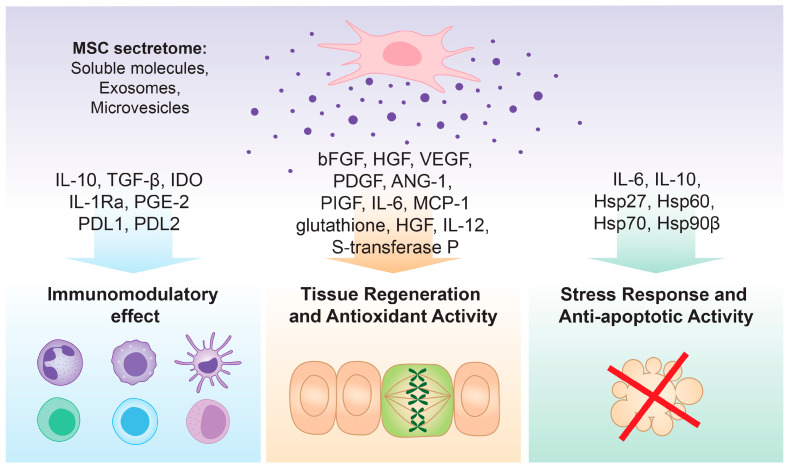
Molecular mechanisms behind the effect of MSC-derived secretome.

The use of cell-free therapies in regenerative medicine (Figure 1) has several advantages over conventional stem cell-based cell therapies [19]: (1) secretome does not contain cells—there is no need to test the donor and recipient to avoid rejection reactions; (2) secretome can be stored without the need for using toxic cryopreservatives, such as Dimethyl Sulfoxide (DMSO); (3) cell-free therapies are safer as they are devoid of viable cells and, therefore, there is no risk of tumor formation and transmission of infections; (4) there is no risk of immunological reactions or zoonotic transmission as fetal bovine serum (FBS) is not used; (5) secretome can be produced in large quantities, lyophilized, packaged and transported more easily and thus be immediately available for treatment, making it a suitable application in emergency cases such as myocardial infarction, cerebral ischemia, and trauma; and (6) lower financial costs associated with maintaining the cell lines.

### 4.1. Immunomodulatory Effect

Mesenchymal stem cells are well known for their immunomodulatory properties. MSCs act as sensors and regulators of inflammation, expressing many immune modulators under different conditions [20]. Their signals can have different and even opposite effects, promoting inflammation when the immune system needs activation and suppressing inflammation when the immune system is already overactivated [21]. Some of the factors are expressed both in the resting and activated state, including PGE2, iNOS, TGFβ, IL-10, HGF, CD39 and CD73, galectins, CCL2, TSG6 (mainly found when MSCs are cultured as spheres), and IL1RA. Others are expressed only in the activated state, including IDO, PD-L1 and PD-L2, and proteins related to the complement system. In contrast, HO-1 is expressed mainly in the resting state and decreases sharply when MSCs are activated. However, all of them are regulated by proinflammatory factors in a concentration-dependent manner. Data indicate that low levels of PGE2 and HLA-G have proinflammatory effects, while high levels have anti-inflammatory effects [20]. In most cases, however, the immunomodulatory activity of MSCs is anti-inflammatory and immunosuppressive, preventing hypersensitivity reactions [22]. By providing relevant signals to immune cells, MSCs can ameliorate the tissue damage caused by autoimmune processes or hypersensitivity reactions associated with infection, particularly viral infection [23]. The immunomodulatory activity of MSCs is achieved via contact-dependent and paracrine signaling, with the latter, at least partly, based on exosomes that bind to recipient cells and can be internalized by them [24]. Researchers have discussed an experimental approach to using exosomes instead of MSCs to treat autoimmune diseases. This concept aims to overcome challenges in traditional cell therapy and avoid transplanting live cells [25,26]. These challenges include the possible formation of blood clots, cell rejection, excessive cell growth, and malignant transformation. Moreover, secretome components are easier to manipulate, store, and package than MSCs themselves [27].

Although direct cell–cell contact is important for the immunosuppressive effects of MSCs, multiple studies have shown that immunomodulators secreted by MSCs are of critical importance. These include indoleamine 2,3-dioxygenase (IDO), prostaglandin E2 (PGE2), iNOS, transforming growth factor-beta (TGFβ), interleukin-10 (IL-10), hepatocyte growth factor (HGF), human leukocyte antigen-G (HLA-G), galectins, C-C motif chemokine ligand 2 (CCL2), and heme-oxygenase 1 (HO-1) [28].

### 4.2. Tissue Regeneration and Antioxidant Activity

Growth factors secreted by MSCs that support angiogenesis and wound healing include basic fibroblast growth factor (bFGF), hepatocyte growth factor (HGF), vascular endothelial growth factor (VEGF), platelet-derived growth factor (PDGF), angiopoietin 1 (ANG-1), placental growth factor (PIGF), interleukin 6 (IL-6), and monocyte chemoattractant protein 1 (MCP-1) [29]. Some studies have investigated the mesenchymal stem cell secretome’s antioxidant properties. By releasing molecules such as glutathione, HGF, IL-12, and S-transferase P, MSCs help counter oxidative stress in damaged tissues [30,31].

### 4.3. Stress Response and Anti-Apoptotic Activity

By inducing STAT3 nuclear translocation and reducing reactive oxygen species (ROS), MSCs can postpone the death of lymphocytes and neutrophils via an IL-6-mediated mechanism [32]. It has also been shown that islet beta cell apoptosis is inhibited by the secretome of mesenchymal stem cells through an IL-10-dependent mechanism [33,34].

Various studies have shown that heat-shock proteins (Hsps) can be a component of mesenchymal stem cell exosomes. These proteins can be secreted into the extracellular environment, where they exert cytoprotective effects. Hsps modulate immune responses and reduce inflammation caused by heat shock. Studies show that Hsps such as Hsp70 protein 5 (HspA5), Hsp70 protein 8 (HspA8), and Hop (Stip1) are highly expressed across various stem cell types (neural stem cells, MSCs, and ESCs), while others, like Hsp70 protein 4 (HspA4), Hsp27 (HspB1), and Hsp90β (HspCb) chaperones, are specific to embryonic stem cells [35,36]. These proteins help maintain stem cell “stemness” by buffering against stress. Additionally, certain Hsps, like Hsp27 and Hsp70, are prominent in mouse embryonic stem cells [37]. Differentiation in human adipose-derived stem cells enhances the expression of Hsp20, Hsp27, Hsp60, and αB-crystallin, highlighting the dynamic role of Hsps in stem cell function [38].

In the context of MSC anti-apoptotic activity, a particular protein deserves special attention: the small heat shock protein Hsp27, also known as HspB1. It is a chaperone that recognizes and binds misfolded proteins, thereby preventing their further denaturation and aggregation, and handles them for refolding to larger chaperones with ATPase activity, such as Hsp70. Furthermore, there is evidence that Hsp27 can influence a number of cellular processes that have an effect on both cellular and organism levels. It increases cell survivability, impacts cytoskeletal dynamics [39], has a role in cellular differentiation, including MSC-triggered ossification [40], shows an inflammatory response [41], and its expression abnormalities are linked to a number of diseases ranging from irritable bowel syndrome [41] to cancer [42]. Hsp27 increases the stress tolerance of cells and has anti-apoptotic activity [43]. Wei et al. prove that Hsp27 and its phosphorylation level have a direct effect on breast cancer cell survivability, cancer stem cell generation, and epithelial-mesenchymal transition [42].

The relation of Hsp27 to MSC has two aspects: its expression and role in MSCs themselves and its possible upregulation in other cells under the influence of MSCs. Hsp27 is expressed in mesenchymal stem cells, especially under stress, and has been found to increase their survival rates. Various researchers have tried to improve the survival and therapeutic potential of MSCs by stimulating Hsp27 expression. The stress-inducing treatments that have been used for this purpose include heat shock [44], visible light-inducing reactive oxygen species [45], and hypoxic pre-conditioning [46]. However, in addition to the potential direct negative effects of stress, there are data that it can cause lasting changes in the gene expression pattern of MSCs far exceeding the induction of heat shock proteins [47]. In a study of MSC therapy in a rat model of myocardial infarction, the long-term specific elevation of Hsp27 levels without inducing stress has been achieved by exogenous expression using a lentivirus vector and has been shown to improve the survival of both the MSCs and the heart tissue in which they were injected [48]. The latter finding is in agreement with the concept that MSC therapy is expected to be effective mostly through secretome-mediated beneficial effects of MSCs on the damaged tissue and, to a lesser degree, by populating the tissue with MSC-derived cells. This raises the question of whether other instances of the MSC-stimulated repair of injured tissue also involve the induction of Hsp27 expression by MSC secretome.

## 5. Mesenchymal Stem Cell Secretome for Treatment of Infertility

Infertility is a global health issue affecting an estimated 17% of people worldwide, with approximately 48 million couples and 186 million individuals experiencing difficulties in conceiving at some point in their lives [49]. Infertility affects both men and women, and the reasons behind it can be very complex (medical, genetic, and environmental) and often even unexplained [50]. The condition often leads to severe psychological, social, and financial challenges, making it a global health issue despite advances in assisted reproductive technologies.

### 5.1. MSC Secretome for Treatment of Female Reproductive Disorders

Female infertility can be caused by blocked or surgically removed fallopian tubes, ectopic pregnancy, untreated sexually transmitted infections, unsafe abortions, or pelvic surgery. Quite often, uterine disorders such as endometriosis, recurrent implantation failure (RIF), congenital abnormalities, Asherman’s syndrome, or fibroids lead to difficulty in conceiving. Other major contributors are ovarian disorders, such as polycystic ovary syndrome (PCOS), premature ovarian failure/insufficiency (POF/POI), and endocrine system disorders impairing hormonal balance, including pituitary cancers or hypopituitarism. These conditions interfere with the proper functioning of the reproductive system, making conception a challenge [49].

#### 5.1.1. Endometrial Damage and Receptivity

The human endometrium is a constantly renewing structure that is under continuous hormonal control, with endometrial remodeling occurring in about 400 cycles during the female reproductive period, consisting of regeneration, differentiation, and shedding. Structurally and functionally, the human endometrium is divided into a basal and a functional layer. The monthly changes in the uterine lining occur with the participation of three types of stem cells: epithelial stem cells (EpSCs), mesenchymal stem cells (MSCs), and endothelial progenitor cells (EPCs) [51].

During the so-called “implantation window”, a period of just a few days in each menstrual cycle, the endometrium is receptive to the embryo. Certain changes in the endometrium allow implantation to occur: increased vascularization, changes in the secretory glands, and expression of certain adhesion molecules (integrins) [52]. However, even the best-quality embryos would not lead to a successful pregnancy if the endometrium receptivity is compromised due to atrophy, inflammation, or immune imbalance [53].

Endometrial stem cells (EnSCs) can be isolated from the endometrium and even from menstrual blood [54,55]. These cells have been extensively studied and characterized as mesenchymal stem cells in accordance with the general criteria of the International Society for Cellular Therapy [56]. EnSCs can be routinely isolated from samples obtained during common gynecological procedures like endometrial scratching or biopsy, and together with other types of mesenchymal stem cells, have been used in different therapies and animal models for the treatment of conditions compromising endometrial receptivity [57].

**Figure 2:**
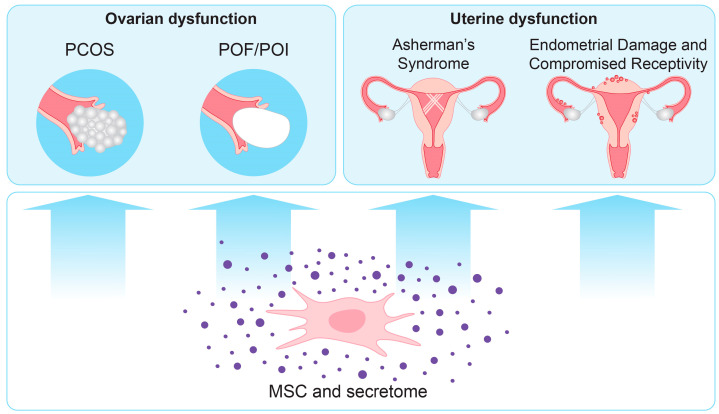
MSC secretome for treatment of female reproductive disorders.

Several studies have examined the effect of MSCs from different tissue sources on endometrial regeneration and receptivity using animal models (Figure 2). MSCs can achieve their regenerative and immunomodulatory effects through paracrine or endocrine secretion of cytokines and growth factors, such as EGF, FGF, PDGF, VEGF, TGFβ, HGF, insulin-like growth factor-1 (IGF-1), SDF-1 (stromal cell-derived factor-1), and angiopoietin-1. Most of these are activated in an NF-κB-dependent manner by inflammatory stimuli, such as IFNγ, TNFα, lipopolysaccharides, or hypoxic conditions [58]. In a study on damaged endometrium using a rat model, bone marrow-derived MSCs (BM-MSCs) were used. They migrated to the site of the damage, where they secreted FGF, which led to enhanced cellular proliferation and increased angiogenesis and implantation rate [59]. Extracellular vesicles (EVs) from BM-MSCs containing miR-340 downregulated collagen 1α1, α-SMA, and TGFβ1 expression, attenuating fibrosis in rats with endometrial damage [60]. In addition, exosomes increased epithelial proliferation, endometrial thickness, and stromal cell migration in a mouse model of endometrial fibrosis. It was shown that miR-29a-3p in the exosomes had an anti-fibrotic effect [61]. A conditioned medium from menstrual blood MSCs enhanced migration, angiogenesis, and proliferation while inhibiting H_2_O_2_-induced apoptosis in HUVECs, through activating AKT and ERK pathways [62]. Several studies have explored the effect of UCMSCs and their exosomes (UCMSCs-exos) on human endometrial stromal cells (hEnSCs) and epithelial cells [63,64]. UCMSCs-exos improved damaged stromal cells [65] and endometrial epithelial cells [66] by downregulating Cleaved Caspase-3 and raising Bcl-2 levels, as well as by activating the PTEN/AKT signaling pathway to control proliferation and prevent apoptosis. In addition, UCMSCs-exos reduce fibrosis markers (α-SMA1 and COL1A1), which, in turn, decrease TGFβ1-induced fibrosis in hEnSCs. ZEB2, a transcription factor that promotes fibroblast development and epithelial–mesenchymal transition (EMT), is downregulated by miR-145-5p [65]. It has been shown that the miR-140-3p/FOXP1/Smad axis is involved in the anti-fibrotic effect of UCSMCs-exos [67]. Exosomes from BM-MSCs that were infected with GFP-CTF1 adenovirus to induce the overexpression of the cytokine Cardiotrophin-1 (CTF1) were used. Treatment increased the migratory, proliferative, and angiogenic potential of HUVECs in a rat model. The exosomes also enhanced endometrial proliferation and angiogenesis, leading to better implantation outcomes [68]. When hydrogels with UCMSCs [69] or human placenta-derived MSCs in combination with hyaluronic acid gels [70] were used, endometrial thickness was significantly increased with a higher implantation rate and elevated VEGF, which stimulated endothelial cell angiogenesis, further confirming the paracrine effects of MSCs. The proliferation of endometrial stromal cells was promoted through the JNK/Erk1/2-Stat3-VEGF pathway and, likewise, glandular cell proliferation through the c-Fos-VEGF and Jak2-Stat5 pathways [70]. Altogether, these studies shed light on the potential processes by which MSC secretome could aid in endometrial healing and lead to better reproductive outcomes [71,72].

#### 5.1.2. Asherman’s Syndrome

Asherman’s syndrome (AS) is a gynecological condition characterized by a damaged basal layer of the endometrium, leading to intrauterine adhesions, hypomenorrhea, and infertility [73]. The most common reason for AS is previous curettage after an abortion. The adhesions formed in the uterus interfere with blastocyst implantation, leading to recurrent miscarriages and implantation failure. Often, the condition remains undetected through routine examination, and despite extensive research, effective treatments remain unavailable.

A number of studies have used various types of hydrogel gels in combination with different types of MSCs and their secretomes to treat intrauterine adhesions (IUAs) in animal models. In a rat model of AS, secretome from MSCs loaded on crosslinked hyaluronic acid gel was used as an approach to repair damaged endometrial morphology [74]. The treatment combination led to significantly more fetuses [75] and reduced the expression of pro-inflammatory factors (IL-1β and IL-6). The secretion of anti-inflammatory cytokine IL-10 was increased, and through the activation of the MEK/ERK1/2 signaling pathway, endometrial VEGF expression was induced, which overall led to higher endometrial receptivity and a higher embryo implantation rate [76]. Using hydrogels loaded with UCMSCs promoted the recruitment of macrophages to the site of the damage and induced their polarization into the M2 phenotype [77,78]. In a recent study, hydrogels embedded with exosomes from adipose tissue-derived MSCs were used, which resulted in HUVEC proliferation and tissue regeneration and higher endometrial receptivity and overall presented an effective system for controlled exosome release for fertility treatments [79].

#### 5.1.3. Premature Ovarian Failure (POF)/Premature Ovarian Insufficiency (POI)

Premature ovarian failure (POF) or premature ovarian insufficiency (POI) is a condition characterized by the loss of normal ovarian function before the age of 40. As a consequence, patients experience lower estrogen production, irregular or even absent menstrual periods, and often, infertility [80]. The etiology includes certain genetic factors, such as Turner syndrome, premutation for fragile X syndrome, and mutations in the genes involved in ovarian function (FOXL2 and BMP15), as well as autoimmune disorders and chemotherapy. Unfortunately, the condition is often idiopathic, and the cause remains unknown.

Studies have shown that mesenchymal stem cells can improve ovarian function through their paracrine effects [81,82] (Figure 2). The primary causes of the reduction in ovarian function are granulosa cell (GC) aging and apoptosis, as the ovary’s GC state is crucial for follicle growth. The inflammatory response was directly linked to the incidence of POI, as evidenced by the elevated expression of pro-inflammatory factors, which makes the use of the MSC immunomodulatory effect a promising treatment path. By secreting vascular endothelial growth factor (VEGF), MSCs stimulated the proliferation of granulosa cells (GC) and oocytes in damaged rabbit ovaries [83]. In a rat model, human UCMSCs restored ovarian function by inducing the secretion of insulin-like growth factor-1 (IGF-1), VEGF, and hepatocyte growth factor (HGF) [84]. Menstrual-derived stem cells secreted fibroblast growth factor 2 (FGF2), which had protective effects on damaged ovaries [85]. In addition, in a mice model of natural ovarian aging (NOA), MSCs boosted ovarian function by secreting EGF and HGF, which slowed oocyte aging and promoted cell proliferation [86]. Exosomes from human UCMSCs that carry miR-126-3p encouraged angiogenesis and reduced the apoptosis of rat ovarian granulosa cells damaged by cisplatin. The study demonstrated that when miR-126-3p UCMSCs–exosomes were administered in a rat model of POF, reproductive organ weights and follicle counts increased, FSH levels decreased, and E2 and AMH levels rose [87]. Human UCMSC-derived exosomes improved ovarian function and reproductive capacity in POI rats by promoting granulosa cell (GC) proliferation and reducing GC apoptosis [88]. Injection of UCSMCs-exos into a chemotherapy-induced POI rat model restored the estrous cycle, normalized sex hormone levels, increased healthy follicles, and reduced atretic follicles [89]. Further research indicates that MSCs inhibit GC ferroptosis. Following treatment with exosomes, granulosa cells’ levels of ROS generation, free iron ions, and lipid peroxidation decreased. Interestingly, hUCMSCs use the stress-response regulator NRF2 to trigger a protective antioxidant pathway in GC [90,91]. Studies have shown that in POI, the expression of SMAD3 in GC is significantly reduced. Human adipose-derived MSC exosomes have been demonstrated to normalize sex hormone levels and restore follicle counts throughout all developmental stages, possibly via lowering apoptosis by inhibiting Fas, FasL, Caspase-8, and Caspase-3 [92]. The phosphorylation of SMAD3 is believed to be involved in the activation of genes linked to follicular growth and development [93]. Several studies have explored the use of exosomes derived from menstrual blood MSCs for the treatment of POI, as the collection of these cells is much less invasive [94,95]. TSP1-containing exosomes enhanced SMAD3 phosphorylation, activating the PI3K/AKT signaling pathway and thereby improving GC function both in vitro and in vivo [96]. The phosphorylation of SMAD3 was significantly increased following treatment with exosomes, which also increased the expression of BCL2 and MDM2 and decreased that of Caspase-3, Caspase-8, p53, and BAX [96]. Altogether, these findings suggest that MSCs from different tissue sources and their secretomes have great potential and multiple advantages as a new approach for the treatment of POI patients and a fertility protection tool [97].

#### 5.1.4. Polycystic Ovary Syndrome (PCOS)

Polycystic ovary syndrome (PCOS) is a very common endocrine disorder among women of reproductive age. It is characterized by irregular menstrual cycles, the presence of cysts in the ovaries, inflammation and hormonal imbalance, including elevated androgen levels, and infertility. Recently, MSCs have been reported as a treatment of PCOS in connection with the search for possible secretome components with therapeutic effects [98,99,100]. MSC secretome has therapeutic effects in PCOS animal models through immunomodulation [101]. In a recent study using a letrozole-induced PCOS mice model, it was found that MSC secretome reversed PCOS-related morbidities, such as insulin resistance and infertility. It also regulated androgen production, inflammation, fat metabolism, and ovarian function [102]. Research suggests that MSCs communicate with ovarian tissue primarily via secretomes and exosomes [19]. UCMSCs can improve ovarian dysfunction in PCOS patients through immunomodulation and inhibition of the NF-κB signaling pathway [103]. In another study, exosomes from adipose tissue-derived MSCs were used in a rat model of PCOS. The exosomes activated the IRS1/AKT pathway and increased hepatic metabolism by transferring miR-21-5p to the livers of rats with PCOS, which suppressed the expression of B-cell translocation gene 2 [104].

### 5.2. MSC Secretome for Treatment of Male Reproductive Disorders

**Figure 3:**
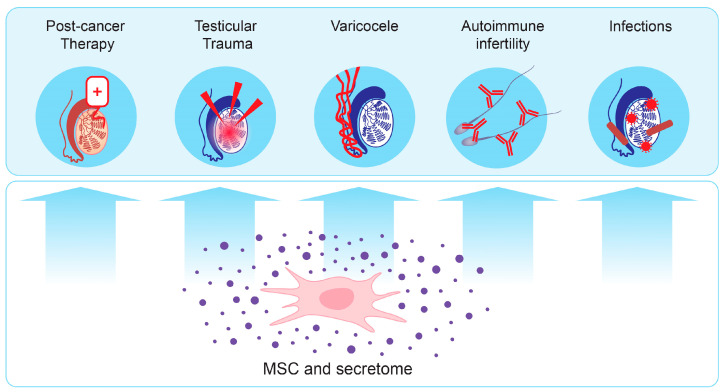
MSC secretome for treatment of male reproductive disorders.

Male subfertility and infertility can have a wide range of causes, including idiopathic, acquired, and congenital conditions. Most of the underlying factors cause testicular damage that affects spermatogenesis, resulting in abnormal sperm quality [105]. In severe cases, not only is natural conception highly unlikely, but also traditional methods of assisted reproductive technology (ART) have a low success rate. On account of their self-renewal, differentiation capabilities, and immunomodulatory effects, stem cell-based approaches have raised great hope in the treatment of male reproductive system disorders by ameliorating their root causes [106] (Figure 3).

#### 5.2.1. Testicular MSCs and the Potential of MSCs to Restore Spermatogenesis

Although mesenchymal stem cells are not listed in the standard descriptions of testicular cell populations, their presence in the testis has recently been reported. Under suitable culture conditions, colonies of mesenchymal stem cells have been obtained from adult human testes removed as part of prostate cancer treatment, as well as testicular biopsies initially taken for diagnostic purposes [107] or for use in assisted reproduction [108]. The authors of the latter study have described the secretion of microvesicles by cultured testicular MSCs and their internalization when added to renal collecting duct cells.

At present, little is known about testicular MSCs and their functions. The methods of their isolation, based on enzymatic treatment of a piece of testicular tissue, do not provide information about their precise localization in vivo. The only somatic cell type known to localize with male germ cells within the seminiferous tubules are Sertoli cells, which play a role analogous to that of granulosa cells in ovarian follicles. It should be noted that embryonic Sertoli cells are mesenchymal before undergoing epithelial transition during the formation of testis cords. In this respect, they resemble MSCs, which are also mesenchymal cells of mesodermal origin. The paracrine secretory profiles of the two cell types are also similar [109]. It could be hypothesized that these common features allow testicular MSCs to contribute to a suitable microenvironment for spermatogenesis by secreting paracrine signals acting either on Sertoli cells or directly on germ cells.

Research on the potential use of MSCs to treat male infertility is focused on their anti-inflammatory, anti-apoptotic, and immunosuppressive activity that could help restore spermatogenesis in testes damaged by toxins, physical factors (trauma or irradiation), infection, or autoimmunity. The majority of published research on the impact of MSCs on spermatogenesis focuses on MSCs from other organs rather than testicular MSCs, as these cells have a longer research history, a better-studied phenotype, and established isolation and culture procedures. The usual approach is the addition of MSCs of a non-testicular origin to the testis or a testicular organ culture and observation of the effects on male germ cells. Numerous studies of this type have found that mesenchymal stem cells have the capacity to stimulate the survival, proliferation, and differentiation of spermatogenic cells, though it is unclear whether this effect is direct or exercised through Sertoli cells.

#### 5.2.2. Improving the Reproductive Potential of Cancer Survivors

Cancer treatment, while preserving the patient’s life, can dramatically decrease his reproductive chances by decimating the germ cell pool, resulting in abolished spermatogenesis. For adult patients, spermatozoa can be cryopreserved. There are reports that the treatment of cryopreserved sperm cells with MSC-derived exosomes improves their post-thaw parameters and increases their fusogenic properties [110]. For childhood cancers, however, the option to collect and preserve sperm cells does not exist. Cryopreservation of testicular tissue with subsequent in vitro maturation has been discussed but is still an experimental approach [111,112,113]. In this respect, there is interest in the potential of MSCs to improve spermatogenesis in preserved testicular tissue under co-culture conditions. In a mouse model, bone marrow-derived mesenchymal stem cells, when co-cultured with tissue from a neonatal mouse testis, have promoted the maturation of Sertoli cells, proliferation of spermatogonial stem/progenitor cells, and their differentiation to the spermatid stage [109]. Some authors used treatment with the alkylating agent busulfan as an approach to induce infertility in animal models in order to mimic the human testis damaged by cancer chemotherapy and have investigated the potential of MSCs and their secretomes to improve spermatogenesis. Human amniotic mesenchymal stem cells, after injection into the testes of busulfan-treated adult mice, have induced restoration of spermatogenesis, testicular weight and size, and testosterone levels [114]. A similar improvement has been obtained by injecting stem cell-derived exosomes into the testes of busulfan-treated prepubescent mice [115]. The secretome effect includes both the improvement of spermatogenesis and a delay in the apoptosis of the spermatogenic cells, even though the mechanism behind it is yet to be explained [116]. In addition to the suppression of apoptosis, a reduction in oxidative stress has been found [117]. In a study using human Wharton’s jelly MSC secretome in a mouse model and investigating sperm DNA maturity, the DNA fragmentation index (DFI), and testicular gene expression, all parameters showed improvement in sperm quality [118].

#### 5.2.3. Improving the Outcome of Testicular Trauma

Methods to treat the impact of trauma on the testis have been studied in rodent models subjected to testicular torsion–detorsion. It has been found that the resulting damage is ameliorated by the intratesticular injection of mesenchymal stem cells [119] or their secreted products [111,120,121]. Human amniotic membrane-derived mesenchymal stem cell-secreted factors reduced oxidative stress, inflammatory response, and apoptosis in mice by modifying the sirtuin-1 nuclear factor erythroid 2-related factor, which, in turn, reduced testicular torsion/detorsion (T/D) damage. The antioxidative, anti-inflammatory, and antiapoptotic qualities of the substances produced by hAMSCs led to an increase in testosterone levels, spermatogenesis, and sperm quality [27]. In another experiment on the influence of MSC secretome, the proliferation and differentiation of rat spermatogonial stem cells were observed in an in vitro co-culture with Sertoli cells and conditioned medium from adipose tissue-derived MSCs [112].

#### 5.2.4. Treatment of Varicocele

The most prevalent medically curable cause of infertility in men is varicocele. Men suffering from varicocele have lower sperm motility and concentration. Even after undergoing varicocelectomy, a considerable proportion of men with clinical varicocele and abnormal semen characteristics remain infertile. Injection of a conditioned medium from adipose-derived mesenchymal stem cells in a surgically created varicocele model has been found to improve spermatogenesis by having an anti-inflammatory and regenerative effect [113].

#### 5.2.5. Improvement of Spermatogenesis in the Aging Testis

Intratesticular injection of exosomes secreted by human umbilical cord mesenchymal stem cells has even reversed some of the effects of aging, increasing proliferation and decreasing apoptosis in the testes of 22-month-old mice [122]. Although such an approach is unlikely to be applied to humans in the foreseeable future, it may eventually be considered because a major cause of the global surge of fertility problems is the increasing tendency to postpone reproduction to a later age.

#### 5.2.6. Ameliorating the Impact of Autoimmunity on Fertility

Antisperm antibodies can have a variety of effects on male fertility. The effects that have been most extensively reported include their ability to decrease sperm motility, induce sperm agglutination, hinder sperm penetration of cervical mucus, and prevent sperm capacitation, the acrosome reaction, sperm–egg interaction, and the early phases of embryo development [123].

In efforts to improve the reproductive chances of patients with antisperm autoimmune responses, some possible approaches are based on the anti-inflammatory and immunosuppressive activity of MSCs found in various studies and presumably based on interaction with immune cells [124]. In a mouse model of antisperm autoimmunity induced by the traumatic rupture of the testis, bone marrow-derived mesenchymal stem cells reduced the incidence of antisperm antibody production without even being near the trauma site: they were introduced by intravenous rather than intratesticular injection and were later found in the lungs but not in the testis, implying generalized effects on the immune response [124]. Based on these data, it is reasonable to expect beneficial effects of MSC therapy in cases of male infertility caused partially or entirely by an autoimmune response against the testis or the spermatozoa.

#### 5.2.7. Improving the Outcome After Infection

Acute infection, particularly viral infection, can inflict lasting damage on the testis. In addition to mumps orchitis being a well-known threat to fertility [125], generalized infections can also have an impact, of which the most recently studied case is post-COVID-19 complications. According to the present data, the negative effects of infection on spermatogenesis are based rarely on viruses replicating inside testicular cells and, more commonly, on inflammation and hypersensitivity reactions caused by the response to the infection. MSCs could downregulate these reactions, as discussed above, for antisperm autoimmunity [23].

#### 5.2.8. Possible Mechanisms of MSC Influence on Male Fertility

The mechanisms by which MSCs exercise the previously described effects on male fertility are only beginning to be elucidated. Some of the cited studies [109,111,115,120,122] report that the beneficial effects of MSCs on spermatogenesis are based on their secretions. In the experiment obtaining the differentiation of neonatal seminiferous tubules under the influence of MSCs [109], the two co-cultured cell populations were separated by a thick layer of liquid medium. Taken together, these findings imply that the effects of MSCs on spermatogenesis are at least partly mediated by their secretomes. While the precise components and their targets have not yet been identified, several of the above reports [109,114,115] specifically find beneficial effects of MSCs not only on spermatogenic cells but also on Sertoli cells. As mentioned above, there are also data that exosomes secreted by MSCs can have a beneficial effect on cryopreserved sperm cells [110]. Based on these data, therapeutic strategies are discussed based on the application of MSC-derived exosomes rather than MSCs themselves [26].

As already mentioned in cases of acute injury or chronic damage to the testis, MSCs improve the recovery of spermatogenesis by suppressing apoptosis [117]. This could be an extension of their function in normal spermatogenesis, which requires a precise balance between survival and apoptotic pathways. Major factors of apoptosis, such as the caspase 9 binding apoptotic protease activating factor-1 (Apaf-1), seem to be crucial for the successful maturation of spermatozoa. Knock-out male mice are shown to be infertile due to mass reduction in spermatogonia numbers and a severely limited maturation rate [126].

It is an interesting question whether the mitigating effects of MSCs after testicular damage are partly mediated by the stimulation of Hsp27 expression, as has been found for intestinal epithelium [127]. All testicular cell populations important for spermatogenesis—germ cells, Sertoli cells, and Leydig cells—are normally positive for Hsp27. In germ cells, Hsp27 is abundantly present in spermatogonia and then decreases in parallel with their differentiation to moderate levels in spermatocytes, low levels in spermatids, and absence in spermatozoa. The expression of Hsp27 in testicular cells is decreased in patients with maturation arrest and even more in those with Sertoli cell-only syndrome [128]. The anti-apoptotic activity of Hsp27 in the testis is thought to offer some degree of protection against the damaging influence of stress and aging [129]. This effect is likely to be caused by the downregulation of apoptotic factors in the cytoplasm of Sertoli cells, spermatogonia, and maturing sperm cells. Hsp27 is shown to reduce the rate of apoptosis in cells by the downregulation of caspase 3 activity by binding caspase 9 depending on Hsp27 modifications [130]. Upregulation of Hsp27 expression and/or changing the modification is one of the mechanisms by which MSCs could exercise a beneficial effect on testes subjected to stress.

Currently, the predominant method for MSC application for the treatment of male infertility is by introducing them into the body, usually by intratesticular injection. However, ex vivo applications are also discussed. One of the above-mentioned experiments included the co-culture of MSCs with samples of testicular tissue, including seminiferous tubules [109]. It is possible that in the future, testicular organoids will also be used if the self-renewal and differentiation of male germ cells can be achieved in this type of culture [131].

## 6. Challenges and Future Directions

Despite the fast-growing number of clinical trials involving MSCs, which, according to clinicaltrials.gov (accessed on 15 February 2025), exceeded 1500, the number of trials based on treatments with MSC secretome was just 27. This could be because the relatively new field of MSC secretome research is still in the preclinical phase, with most studies based on animal models. Another possible reason arises from factors like donor specificities, culture medium composition, cell culture passage, storage conditions, and pretreatment protocols, all affecting MSC secretome properties and therapeutic potential [132,133,134].

### 6.1. Variability of MSCs

Despite the widespread belief in the dichotomic paradigm in terms of stem cells, in which the cell is or is not essentially a stem cell, it seems clear that stem cells are characterized by a wide range of properties that depend on many factors. In this case, the heterogeneity of the stem cells between the different stem cell lines can be considered the norm, not an exception, and this variability is associated with several factors, including the peculiarities of the individual donor, the source of the cells, and the culture conditions [135]. Considering the many variables, if we focus specifically on the subgroup of mesenchymal stem cells, there can be many variations in the properties and behavior of cells. This variability complicates the production of consistent MSC products for therapeutic use. Addressing this challenge is crucial for advancing MSC therapies [136].

Several factors, including age, gender, and phenotype, have already been established as potential causes of MSC heterogeneity, which affects MSC functions like angiogenesis, immunomodulation, and regeneration [137,138]. Variability can be introduced by factors like media supplementation, culturing techniques, expansion level, and differences in administration protocols [139]. Han et al. compared four sources of MSCs (bone marrow, fat, umbilical cord, and placenta) of 22 individuals. There was significant variability in proliferative capacity despite the identical methods of cultivation in individual samples [140]. Even within the same individual, there was significant heterogeneity in the four different tissue sources despite the same methodology and conditions. Another study examined the techniques for the isolation of umbilical cord MSCs. The authors found differences in the profile of cellular proliferation between the different techniques [141]. Even when strict protocols were followed, each cord sample had distinct behavior [141]. MSCs obtained from different types of bone marrow aspirates showed variability in concentration and cytokine secretion [142]. One study explored the use of harmonized culture conditions implemented in different laboratories. Although variability between the MSCs was decreased, it was not eliminated [143].

It makes sense that the secretomes of various MSC types will vary given all of these differences, which will inevitably influence their therapeutic potential [144,145]. A comparison of secretomes from MSCs from adipose tissue, bone marrow, placenta, and Wharton’s jelly was performed by mass spectrometry. As expected, the secretome protein profiles shared many common functions, including promoting cell migration, proliferation, and anti-apoptosis. Secretomes from fetal MSCs showed stronger correlations with developmental and metabolic processes compared to adult MSCs, possibly due to their birth-related origins. It should be noted that the study could not take into account the changes in MSC secretome in the context of the specific microenvironment and the presence of inflammation, something the authors also discussed [146]. The inflammatory environment precedes the ability of MSCs to suppress immune responses. IFNγ, in combination with some other pro-inflammatory cytokines, such as TNFα, IL-1α, or IL-1β, may encourage MSCs to secrete high levels of immunosuppressive factors.

### 6.2. Ethical and Regulatory Aspects of Treatments Involving MSCs and Their Secretomes

Therapies involving MSCs and their secretomes, like any new treatment approach, have their ethical and legal implications. A major advantage of MSCs is that, unlike embryonic stem cells, they do not require the destruction of an early human embryo. However, there are still ethical challenges related to the potential risks of MSC therapy. Some clinical trials using MSCs for the treatment of conditions unrelated to infertility have shown complications due to the differentiation of MSCs to unwanted tissues (including ectopic cartilage and bone) or undesired immunomodulation based on their double capacity to suppress or promote inflammation. Studies on mouse models have also revealed a risk of suppressing antitumor immunity and thus accelerating tumor growth [147]. Most of these risks are bypassed when MSC secretome is used instead of MSCs themselves, although there are concerns that in certain circumstances, MSC exosomes could contribute to tumor growth [148].

Both cell-based and cell-free therapy using allogeneic MSCs or their secretomes is subject to the usual ethical and legal constraints of treatments involving donor tissues. This issue is of least concern in the case of umbilical cord-derived MSCs because the umbilical cord is traditionally regarded as a by-product to be discarded; nevertheless, any research or therapeutic use of umbilical cord-derived cells requires proper informed consent of the parents [149].

As MSCs and their secreted products are considered drugs, all procedures related to their collection and manipulation must be ethically approved and conform to the Good Manufacturing Practice (GMP) in the European Union No. 1394/2007 and the Current Good Tissue Practice requirements (GTP) in the United States Code of Federal Regulation (CFR) Title 21 Part 1271 [150]. In addition, cell-free therapy using MSC secretome must follow the minimal information for studies of extracellular vesicles 2018 guidelines of the International Society for Extracellular Vesicles [151].

### 6.3. Lack of Standardization

MSC secretome has gained interest for its therapeutic potential as a cell-free alternative. However, difficulties arise from variations in MSC isolation, characterization, and secretome storage. Regulatory challenges arise from the lack of standardized assays and manufacturing processes. The specific characterization criteria for MSCs are outdated and insufficient, leading to discrepancies in applications. With the increasing number of MSC-secretome possible clinical applications and the need for large-scale manufacturing, standardization is needed to manage product variability and improve therapeutic consistency. Establishing a consensus MSC definition would enhance comparability across studies and facilitate regulatory approval [152,153]. However, some experts warn against strict standards due to existing gaps in our knowledge about MSC biology and the observation that it is normal for mesenchymal stem cells to exhibit different properties depending on their origin and especially the environment in which they are found [154]. Different approaches have been suggested for further MSC standardization, including the use of additional surface antigens, mRNA expression analysis, differentiation assays complemented with specific gene expression profiles, endothelial cell tube formation assay, and lymphocyte proliferation suppression tests [139]. However, concerning MSC secretome, the difficulties associated with its standardization become even greater, where any change in the manufacturing approach will change the final product [132].

### 6.4. Future Directions

Over the past few decades, pretreatment of MSCs before application has been implemented to boost the immunomodulatory efficacy of MSC therapy. To improve the immunomodulatory and regenerative effects of MSCs, a variety of pretreatment techniques have been employed, such as hypoxia, inflammatory and growth factors, hormones, three-dimensional cell culture in the form of MSC spheroids, and pharmacological and chemical agents [155,156,157]. It should be once again emphasized that the standardization of MSC secretome production is crucial for clinical applications [132]. Further research is required to find the optimal MSC type, culture conditions, pretreatment protocols, and secretome content for clinical application [158].

## 7. Conclusions

The increasing variety of experimental techniques and amount of knowledge regarding stem cell secretomes are evidence of the great desire of scientists and clinicians to find therapies and gain a better understanding of the molecular mechanisms within them. MSC secretome holds great promise as a novel therapeutic approach for treating infertility. Its ability to reduce inflammation, promote tissue regeneration, and induce angiogenesis is achieved by the great variety of bioactive molecules it contains, including growth factors, cytokines, Hsp, and extracellular vesicles. Numerous studies have shown that MSC-derived secretomes can induce endometrial regeneration, restore ovarian reserve, and improve spermatogenesis. While further research is required to standardize secretome production and evaluate its long-term safety and efficacy, MSC secretome is, without doubt, a groundbreaking approach in regenerative medicine, providing an individualized and minimally invasive treatment for reproductive disorders.

## Abbreviations

The following abbreviations are used in this manuscript:

MSC	Mesenchymal stem cell
DNA	Deoxyribonucleic acid
RNA	Ribonucleic acid
ESC	Embryonic stem cell
EGC	Embryonic germ cell
HSC	Hematopoietic stem cell
ISCT	International Society for Cellular Therapy
CD	Cluster of differentiation
HLA	Human leukocyte antigen
NK	Natural killer
TNFα	Tumor necrosis factor alpha
IL	Interleukin
IFNγ	Interferon gamma
DMSO	Dimethyl sulfoxide
FBS	Fetal bovine serum
PGE2	Prostaglandin E2
iNOS	Inducible nitric oxide synthase
TGFβ	Transforming growth factor-beta
HGF	Hepatocyte growth factor
CCL2	C-C motif chemokine ligand 2
TSG6	Tumor necrosis factor-stimulated gene 6
IL1RA	Interleukin-1 receptor antagonist
IDO	Indoleamine 2,3-dioxygenase
PD-L	Programmed death-ligand
HO-1	Heme-oxygenase 1
FGF	Fibroblast growth factor
bFGF	Basic fibroblast growth factor
VEGF	Vascular endothelial growth factor
PDGF	Platelet-derived growth factor
ANG-1	Angiopoietin 1
PIGF	Placental growth factor
MCP-1	Monocyte chemoattractant protein 1
ROS	Reactive oxygen species
ATPase	Adenosine triphosphatase
RIF	Recurrent implantation failure
PCOS	Polycystic ovary syndrome
POF/POI	Premature ovarian failure/insufficiency
EpSC	Epithelial stem cell
EPC	Endothelial progenitor cell
EnSC	Endometrial stem cell
EGF	Epidermal growth factor
IGF-1	Insulin-like growth factor-1
SDF-1	Stromal cell-derived factor-1
NF-κB	Nuclear factor kappa B
BM-	Bone marrow-derived
EV	Extracellular vesicles
α-SMA	Alpha-smooth muscle actin
HUVEC	Human umbilical vein endothelial cell
ERK	Extracellular signal-regulated kinase
UC	Umbilical cord-derived
Exos	Exosomes
hEnSC	Human endometrial stromal cell
COL1A1	Collagen type I alpha 1
EMT	Epithelial–mesenchymal transition
GFP	Green fluorescent protein
CTF1	Cardiotrophin-1
AS	Asherman’s syndrome
IUA	Intrauterine adhesions
BMP	Bone morphogenetic protein
GC	Granulosa cell
NOA	Natural ovarian aging
FSH	Follicle-stimulating hormone
E2	Estradiol
AMH	Anti-Müllerian hormone
TSP1	Thrombospondin-1
ART	Assisted reproductive technology
DFI	DNA fragmentation index
T/D	Torsion/detorsion
hAMSC	Human amniotic mesenchymal stem cell
Apaf-1	Apoptotic protease activating factor-1
